# Payer Type and Emergency Department Visit Prices

**DOI:** 10.1001/jamanetworkopen.2024.1297

**Published:** 2024-03-06

**Authors:** Jacob R. Morey, Richard C. Winters, Aidan F. Mullan, John Schupbach, Derick D. Jones

**Affiliations:** 1 Department of Emergency Medicine, Mayo Clinic, Rochester, Minnesota

## Abstract

This cross-sectional study assesses list prices, cash prices, and negotiated rates for emergency department services.

## Introduction

Health care costs are a source of financial hardship for many US residents.^1^ Contributing to this problem is a lack of price transparency, which prevents patients from shopping for health care services and health insurers from negotiating rates.^2^ The Centers for Medicare and Medicaid Services Hospital Price Transparency rule requires hospitals to disclose list prices and negotiated rates for specific services. Among these services are emergency department (ED) visits, which have recently seen a substantial and disproportionate increase in facility fees (80% of ED visit costs).^1,3^ In this study, we compared list prices, cash prices, and negotiated payer rates for the facility fees of ED visits at low-, moderate-, or high-level medical decision-making (billing code levels 3-5), corresponding with *Current Procedural Terminology* (*CPT*) codes 99283 to 99285.

## Methods

This cross-sectional study analyzed billing rates for quarter 4 of 2022 using a publicly available dataset (Turquoise Health). This dataset consists of facility fees disclosed by all hospitals in compliance with the Hospital Price Transparency rule (approximately 5750 of 6330 total hospitals are compliant); numbers of ED visits are not included. The Mayo Clinic Institutional Review Board deemed this study exempt from ethics review and informed consent because it was not human participant research. We followed the STROBE reporting guideline.

*CPT* codes 99283 to 99285 were selected, and prices and rates were categorized as list price, cash price, private insurance, Medicare Advantage, and Managed Medicaid (eMethods in Supplement 1). Prices and rates were recorded directly; no conversions were required. The main outcome was the comparison of cash prices and negotiated rates for private insurance, Medicare Advantage, and Managed Medicaid to list prices for *CPT* codes 99283 to 99285.

Comparisons were performed using log-γ regression and reported as percent differences with 95% CIs. Regression models were both unadjusted and adjusted for hospital state, rating, and size. Two-sided *P* < .05 indicated statistical significance. Statistical analysis was performed using R 4.2.2 (R Core Team).

## Results

A total of 489 847 ED visit facility fees were reported, including 159 232 (32.5%), 161 537 (33.0%), and 169 078 (34.5%) with *CPT* codes 99283, 99284, and 99285, respectively (Table). The median (IQR) facility list prices for these *CPT* codes were $696 ($359-$1292), $1189 ($589-$2106), and $1784 ($863-$3194), respectively (Figure). After adjusting for hospital state, rating, and size, Managed Medicaid rates were the lowest relative to list prices across all complexity levels for *CPT* codes 99283, 99284, and 99285 (−74.8%, −78.4%, and −79.5%; all *P* < .001), followed by Medicare Advantage rates (−72.5%, −72.6%, and −72.7%; all *P* < .001), cash prices (−37.0%, −37.2%, and 39.7%; all *P* < .001), and private insurance rates (−36.1%, −39.0%, and −39.4%; all *P* < .001). There was more variation in list prices, private insurance rates, and cash prices compared with Medicare Advantage and Managed Medicaid rates. Both hospital rating and size were associated with increased prices and rates (3.2% and 19.9%, respectively, for *CPT* code 99285).

**Table.** Summary of Price and Rate Categories, Hospital Characteristics, and Prices and Rates by Emergency Department Billing Code Level

	*CPT* code and billing code level, No. (%)		
	99283, Level 3 (n = 159 232)	99284, Level 4 (n = 161 537)	99285, Level 5 (n = 169 078)
Price and rate categories			
List price	7372 (4.6)	7557 (4.7)	7753 (4.6)
Cash price	6464 (4.1)	6609 (4.1)	6797 (4.0)
Private insurance rate	110 853 (69.6)	111 446 (69.0)	117 589 (69.5)
Medicare Advantage rate	24 993 (15.7)	25 976 (16.1)	26 506 (15.7)
Managed Medicaid rate	9550 (6.0)	9949 (6.2)	10 433 (6.2)
Hospital rating			
1	6278 (3.9)	7115 (4.4)	6732 (4.0)
2	20 725 (13.0)	20 980 (13.0)	22 030 (13.0)
3	32 263 (20.3)	33 097 (20.5)	34 451 (20.4)
4	36 004 (22.6)	36 544 (22.6)	37 439 (22.1)
5	19 903 (12.5)	20 322 (12.6)	21 643 (12.8)
Unknown or not rated	44 059 (27.7)	43 479 (26.9)	46 783 (27.7)
Hospital size (No. of beds)			
≤100	68 348 (42.9)	67 915 (42.0)	71 724 (42.4)
101-250	37 425 (23.5)	38 969 (24.1)	39 469 (23.3)
≥251	51 774 (32.5)	53 187 (32.9)	56 161 (33.2)
Unknown	1685 (1.1)	1466 (0.9)	1724 (1.0)
Price and rate, median (IQR), $			
List price	696.00 (358.86-1291.50)	1189.0 (589.00-2106.30)	1783.50 (863.08-3194.00)
Cash price	420.30 (238.48-729.73)	699.00 (386.87-1235.00)	1058.80 (570.00-1863.20)
Private insurance rate	517.00 (258.55-853.10)	838.00 (417.94-1441.75)	1280.47 (614.55-2239.66)
Medicare Advantage rate	231.60 (208.00-276.00)	367.14 (329.30-443.00)	527.14 (466.01-646.00)
Managed Medicaid rate	178.89 (84.86-282.59)	272.88 (147.06-438.46)	334.97 (197.38-632.00)

Abbreviation: *CPT*, *Current Procedural Terminology*.

**Figure.**
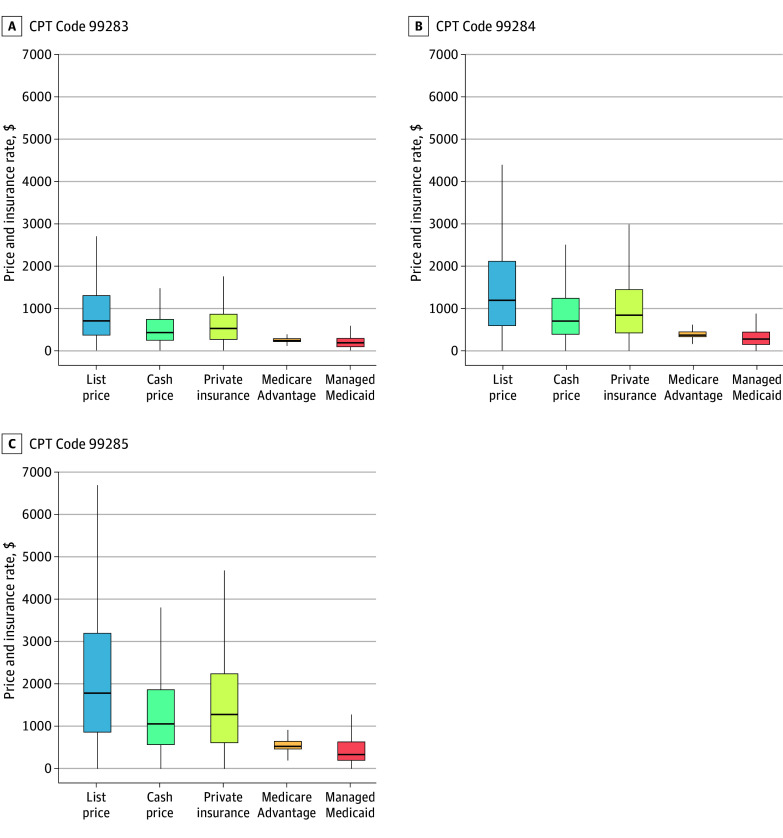
List Prices, Cash Prices, and Negotiated Rates for Private Insurance, Medicare Advantage, and Managed Medicaid Upper and lower ends of the boxes represent upper (75%) and lower (25%) quartiles, respectively; horizontal line inside boxes represent the median; and whiskers represent the minimum and maximum. *CPT* indicates *Current Procedural Terminology*.

## Discussion

In a national comparison to list prices for ED visit facility fees, prices and rates ranked from highest to lowest were for private insurance, cash price, Medicare Advantage, and Managed Medicaid. These list and cash prices were similar to those in previous national studies but significantly lower than in a Florida study.^4,5^ Negotiated rates were also similar to rates in a recent study that was not specific to ED visits; however, that study did not indicate whether Medicare Advantage and Managed Medicaid plans were included.^6^ This information is important given that Medicare Advantage rates are capped at traditional Medicare fee-for-service rates, even if the hospital is out of network.

Study limitations included data quality within the public dataset, hospital compliance with disclosing data, and data availability for quarter 4 of 2022. Future research should explore the role of transparency in changing prices and rates over time, hospital and payer characteristics, more pricing categories, and both affordability and access for patients.

## Notes

Supplement 1.
eMethods.

Supplement 2.
Data Sharing Statement
