# Supplementary material for: Payer Type and Emergency Department Visit Prices
Source: JAMA Netw Open. 2024 Mar 6;7(3):e241297. doi: 10.1001/jamanetworkopen.2024.1297 (PMC10918506; doi:10.1001/jamanetworkopen.2024.1297)
Supplement: Supplement 1. — eMethods. [file jamanetwopen-e241297-s001.pdf]

Supplemental Online Content

Morey J, Winters R, Mullan A, Schupbach J, Jones D. Payer type and emergency department visit prices. *JAMA Netw Open*. 2024;7(3):e241297.
doi:10.1001/jamanetworkopen.2024.1297

eMethods.

This supplemental material has been provided by the authors to give readers additional information about their work.

© 2024 Morey J et al. *JAMA Network Open*

eMethods

The aim was to compare facility fees prices/rates between list prices, cash prices, private insurance, Medicare Advantage, and Managed Medicaid. Facility fees cover the costs of the medical building, utilities, supplies, technologies, and support staff, such as nursing care, technicians, and administrative staff. We refer to the list and cash prices as the ‘prices’ and the negotiated rates between hospitals and private insurance, Medicare Advantage, and Managed Medicaid plans as ‘rates’.

List prices are set by hospitals and are not specific to payer type. Cash prices were reported directly by hospitals and no conversions were required. Private insurance (including employer-based plans and private insurance plans on the Health Insurance Marketplace [HealthCare.gov]), Medicare Advantage, and Managed Medicaid rates were negotiated between payers and hospitals as reported by hospitals. Medicare Advantage and Managed Medicaid plans refer to plans that private insurers offer to individuals eligible for Medicare or Medicaid. Eligibility for Medicare is generally individuals aged 65 or older. Medicaid eligibility varies by state but is generally for low-income families, pregnant women, and children. The dataset does not include rates from Traditional Medicare or fee-for-service Medicaid. The dataset also includes hospital state, CMS Overall Hospital Quality Start Rating (1-5; referred to as ‘hospital rating’), and hospital size (total number of beds: 100 or lower, 101-250, and 251 or higher).

The dataset had complete data for prices/rates and hospitals. There were 4,875 records (1,685 CPT code 99283, 1,466 CPT code 99284, and 1,724 CPT code 99285) missing the number of hospital beds, which were excluded only from the multivariable analysis. Military, government, special needs plans, and dual-eligible insurance plans, as well as any payer type with less than 10 reported rates, were excluded. Outliers in billing rates were addressed by excluding data exceeding the 99th percentile for the specific billing code.

© 2024 Morey J et al. *JAMA Network Open*
