# Supplementary material for: Payer Type and Emergency Department Visit Prices
Source: JAMA Netw Open. 2024 Mar 6;7(3):e241297. doi: 10.1001/jamanetworkopen.2024.1297 (PMC10918506; doi:10.1001/jamanetworkopen.2024.1297)
Supplement: Supplement 2. — Data Sharing Statement [file jamanetwopen-e241297-s002.pdf]

Data Sharing Statement

Morey. Payer Type and Emergency Department Visit Prices. *JAMA Netw Open*. Published March 06, 2024. doi:10.1001/jamanetworkopen.2024.1297

Data
Data available: No
